# Ischemic Retinal Vasculitis and Its Management

**DOI:** 10.1155/2014/197675

**Published:** 2014-04-15

**Authors:** Lazha Talat, Sue Lightman, Oren Tomkins-Netzer

**Affiliations:** ^1^Moorfields Eye Hospital, City Road, London EC1V 2PD, UK; ^2^UCL Institute of Ophthalmology, London EC1V 9EL, UK

## Abstract

Ischemic retinal vasculitis is an inflammation of retinal blood vessels associated with vascular occlusion and subsequent retinal hypoperfusion. It can cause visual loss secondary to macular ischemia, macular edema, and neovascularization leading to vitreous hemorrhage, fibrovascular proliferation, and tractional retinal detachment. Ischemic retinal vasculitis can be idiopathic or secondary to systemic disease such as in Behçet's disease, sarcoidosis, tuberculosis, multiple sclerosis, and systemic lupus erythematosus. Corticosteroids with or without immunosuppressive medication are the mainstay treatment in retinal vasculitis together with laser photocoagulation of retinal ischemic areas. Intravitreal injections of bevacizumab are used to treat neovascularization secondary to systemic lupus erythematosus but should be timed with retinal laser photocoagulation to prevent further progression of retinal ischemia. Antitumor necrosis factor agents have shown promising results in controlling refractory retinal vasculitis excluding multiple sclerosis. Interferon has been useful to control inflammation and induce neovascular regression in retinal vasculitis secondary to Behçet's disease and multiple sclerosis. The long term effect of these management strategies in preventing the progression of retinal ischemia and preserving vision is not well understood and needs to be further studied.

## 1. Background

Retinal vasculitis is a sight-threatening inflammatory condition, occurring in approximately one in every eight eyes with uveitis [1]. Based on the etiology, retinal vasculitis may be classified as either idiopathic or secondary to infection, neoplasia, or a systemic inflammatory disease [2, 3]. In a cohort study involving 1390 patients with uveitis, 15% had retinal vasculitis as part of their uveitic manifestations [1]. The main concern with retinal vasculitis is the risk of developing vasooclusion and retinal ischemia that can lead to serious sight threatening manifestations. In a retrospective study of 113 eyes with retinal vasculitis in eastern India, capillary nonperfusion was the most common fundus fluorescence angiography (FFA) finding seen in retinal vasculitis, found in 40% of the cases, followed by collateral vessels, seen in 19.5% of eyes with vasculitis [4]. Different causes of retinal vasculitis carry variable risks of developing retinal ischemia ranging from being common in presumed tuberculous retinal vasculitis and Behçet's disease to a more rare association in sarcoidosis and multiple sclerosis (Table 1) [3, 5].

**Figure 1:**
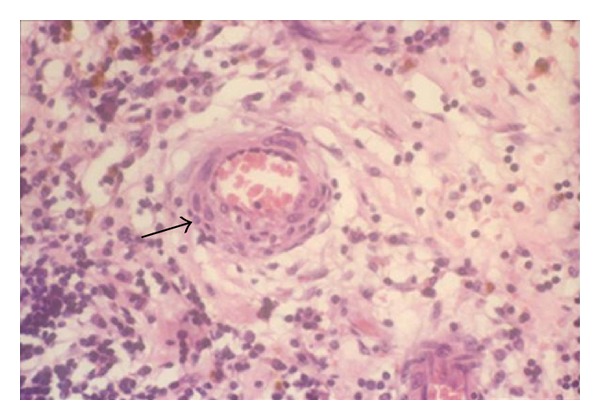
Histopathological image of a retinal blood vessel involved in Behçet's disease (H & E stain). Note the perivascular infiltration of lymphocytes around the vessel (arrow).

The pathogenesis of ischemia in retinal vasculitis is not clear but is suggested to be either thrombotic or obliterative secondary to the infiltration of inflammatory cells (Figure 1). Based on histological studies, vascular changes in uveitis are characterized by perivascular infiltration of lymphocytes resulting in perivasculitis rather than a true vasculitis of the vessel wall [6, 7]. Cell-mediated immunity also plays a role in the pathology of retinal vasculitis, with CD4+ T cells documented within and around the retinal vessels. Thrombotic vascular changes can occur due to local endothelial injury or increased prothrombin activity as observed in Behçet's disease [8]. The retina has a uniquely high metabolic demand for oxygen that is normally met by a highly efficient vascular supply. Insufficiency of the retinal circulation causes neuroretinal dysfunction and degeneration. Focal retinal ischemia results in selective damage to specific subpopulations of retinal neurons and can result in cellular death by apoptosis or necrosis with dysfunction and degeneration of the inner retina and eventually visual loss. Retinal vascular obstruction can also promote the production of vascular endothelial growth factor (VEGF), which increases vascular permeability and results in macular edema and induced neovascularization [9].

The management and long term outcomes of ischemic retinal vasculitis as a whole have rarely been addressed in prospective studies. In one retrospective study, 20 patients (38 eyes) with ischemic retinal vasculitis were compared to 33 patients (62 eyes) with nonischemic vasculitis. While the initial visual acuity was not significantly different between the two groups, 13 (34%) eyes in the ischemic group had final severe visual loss compared with 4 (6%) eyes in the nonischemic group and no significant difference in the median number of relapses/year between both groups [10]. The risk of visual loss in cases with retinal ischemia relates to involvement of posterior pole as in macular edema and macular ischemia or due to stimulating neovascularization (NV) at optic disc (NVD) or elsewhere in the retina (NVE). These fragile new vessels bleed easily resulting in vitreous hemorrhage (VH), fibrovascular proliferation, and subsequent tractional retinal detachment. While the NV itself is managed mainly using scattered laser photocoagulation (SLP) to the ischemic area, the role of immunosuppressive/immunomodulatory (IMS) medications in preventing further progression of retinal ischemia is not fully understood.

## 2. Presumed Tuberculous Retinal Vasculitis

Ischemic retinal vasculitis may be secondary to tuberculous infection (TB) or as a result of a hypersensitivity reaction to tuberculoprotein. In a clinical review of 21 patients with presumed ocular TB infection, occlusive retinal vasculitis was the most common presentation affecting 12 patients, of which eight (38%) had underlying active systemic TB [11]. In another study on 73 eyes (51 patients) with presumed TB uveitis, the authors found retinal periphlebitis in 35% of eyes involved. This was complicated by NV in 29% (half seen on presentation), VH in 11%, and retinal detachment in 3% of eyes [12].

**Figure 2:**
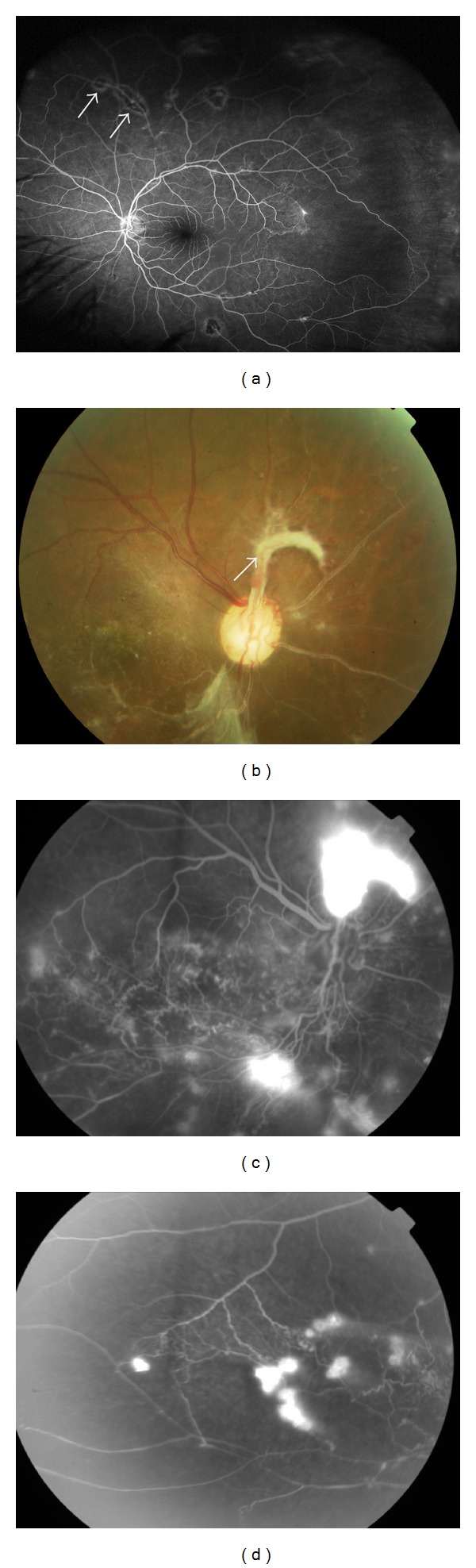
Fundus images of presumed tuberculous occlusive vasculitis. (a) Fundus fluorescein angiography shows peripheral retinal nonperfusion together with small area of hypofluorescence corresponding to chorioretinal lesions along the retinal blood vessels (arrow). (b) A color image showing vascular sheathing together with a fibrovascular tuft originating from the optic disc (arrow), with fluorescein angiography showing (c) leakage at the disc and (d) peripheral capillary dropout and dye leakage from new vessels elsewhere.

Possible mechanisms resulting in venous occlusion include disc edema secondary to tuberculous inflammation or obliteration of the vessels by a hypersensitivity reaction to* M. tuberculosis.* In these cases, occlusive periphlebitis can affect the retina in multiple quadrants and is associated with thick exudates around the retinal veins and retinal hemorrhages. As a consequence to retinal ischemia, NV, VH, traction retinal detachment, rubeosis iridis, and neovascular glaucoma can occur [5]. CRVO has also been reported [13, 14] and may be associated with retinal vasculitis, chorioretinitis, and retinal ischemia. In one case, the inflammation resolved gradually following the initiation of anti-TB therapy, while intravitreal bevacizumab therapy given one month after presentation had little effect with VH occurring five months after the injection [14]. Presumed TB retinal vasculitis can result in extensive peripheral capillary closure with recurrent VH in young adult males, in the absence of other features of intraocular inflammation such as vitreous cells. In other cases, active or healed patches of focal choroiditis along the retinal veins can help to differentiate presumed TB vasculitis from other causes of retinal vasooclusion (Figure 2) [15].

## 3. Behçet's Disease

Ocular involvement in Behçet's disease (BD) occurs in approximately 70% of the patients and is associated with a high risk of visual loss [16, 17]. In a retrospective study of 107 patients with ocular BD, the 10-year risk of developing severe visual loss of 6/60 or worse was 13% and ischemic maculopathy secondary to BRVO was attributed to half the cases of irreversible severe visual loss [18]. The contribution of BD on the overall incidence of retinal vasculitis can vary based on the population at risk. A review of 1390 uveitis cases on the west coast of the United States found 207 patients with evidence of retinal vasculitis; of these cases, only 14 patients had BD [1]. On the other hand, retinal vasculitis is common among patients with ocular BD. In one multicentre study, 22% of eyes with ocular BD had retinal vasculitis [16].

**Figure 3:**
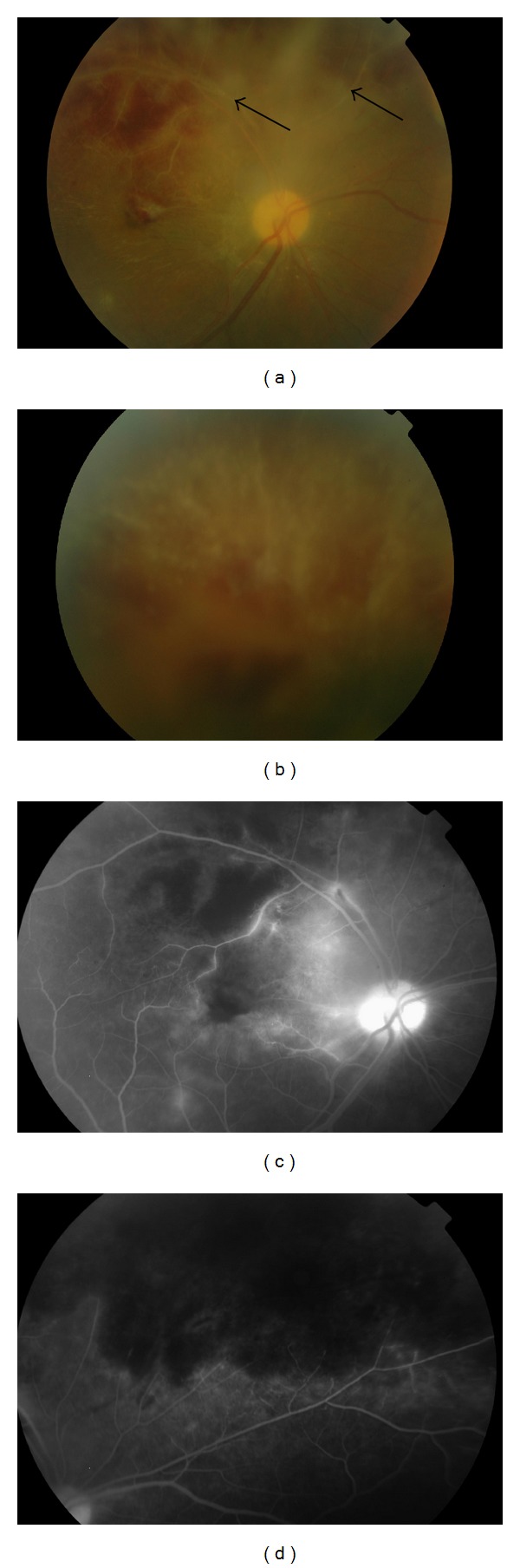
Fundus photographs of branch retinal vein occlusion secondary to Behçet's disease. (a, b) Color images of the right eye showing vascular sheathing (arrows), exudates, and intraretinal hemorrhages. (c) Fluorescein angiography demonstrates multiple areas of hypofluorescence corresponding to areas of retinal hemorrhage and (d) upper retinal quadrant hypoperfusion secondary to vasooclusion.

Retinal vasculitis in ocular BD most commonly manifests as vitritis with diffuse vascular leakage on FFA due to inflammatory hyperpermeability. This may be accompanied by capillary nonperfusion secondary to occlusive vasculitis resulting in NV. Both retinal arteries and veins can be involved in BD though venous involvement is more common [3]. BRVO with intraretinal hemorrhages and macular edema are frequently seen and these are often central in the retinal with a high risk of significant visual loss (Figure 3). BRVO and ischemic retinal vasculitis have been reported as the first presentation of ocular BD in 28% and 21%, respectively, while central vein (4%) and artery (1%) occlusions are less common presentations [18]. Macular ischemia, a predictor of poor visual outcome, has also been reported in cases with BD. In a recent retrospective study of 120 eyes of patients with BD, macular ischemia was seen in one eye (0.8%) at initial visit, while three eyes (2.5%) developed ischemia during a mean follow-up period of 22 months [19]. NV is a serious complication observed by one study in 4% of 1567 eyes with Behçet's uveitis [20], and a multicentre study reported an incidence rate of 0.12 to 0.17 per person per year [16]. NV in BD can be secondary to inflammation and regress in response to IMS therapy or present as an early complication of Behçet's uveitis even in the absence of retinal ischemia [21].

## 4. Systemic Lupus Erythematosus 

The incidence of retinopathy in patients with systemic lupus erythematosus (SLE) ranges from 3% to 29% [22–24] depending on the studied population and associated risk factors for SLE retinopathy such as the presence of anticardiolipin antibodies, central nervous system involvement, serum creatinine level, and SLE activity [22, 25]. Retinal vasculopathy and associated vascular occlusion are a sight threatening manifestation of SLE retinopathy, reported to cause severe visual loss in 55% of patients [26]. The main factor affecting visual outcome in these cases is the occurrence of NV with or without VH, reported in about 40% of the cases [23], as well as an increased risk of developing retinal vein occlusion [27]. Vasoocclusive retinopathy can be the primary manifestation that leads to the diagnosis of SLE [28].

The exact pathogenesis of vascular occlusion is not clear, but there have been proposed theories on the role of immune-complex deposition and complement activation with fibrinoid degeneration of the vascular wall as factors contributing to the vascular damage seen in these cases [29, 30]. Occlusive retinal vasculopathy involving the retinal arterioles may present with cotton-wool spots, predominantly in the posterior pole, representing retinal microinfarctions.

**Figure 4:**
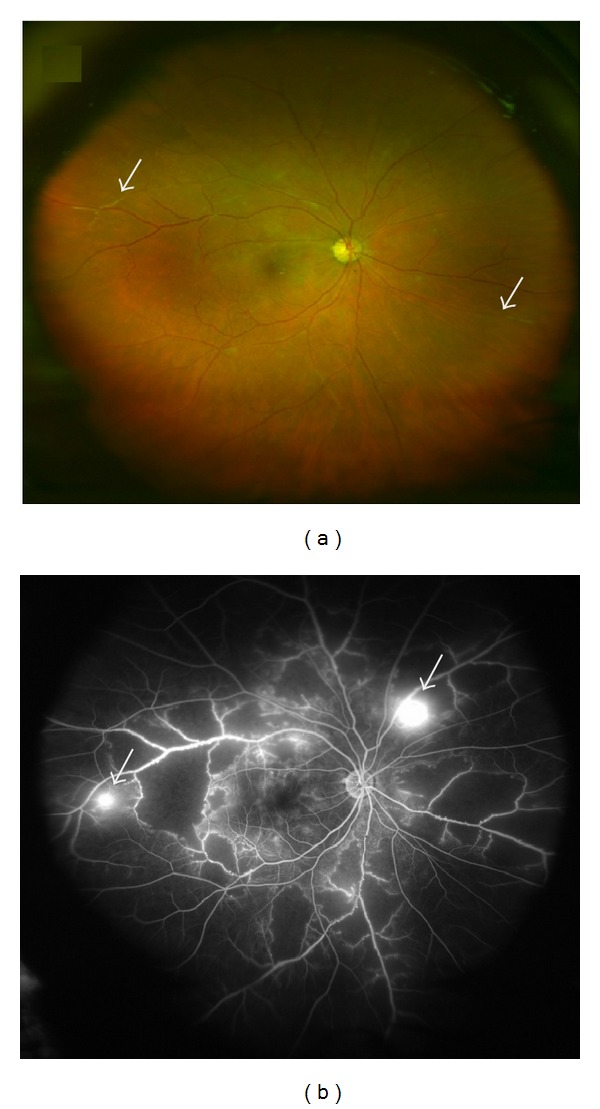
Fundus photographs of SLE associated occlusive retinal vasculitis. (a) Color images demonstrating vascular sheathing (arrows). (b) Fluorescein angiography shows multiple areas of capillary dropout at the retinal midperiphery with leakage from retinal neovascularization (arrows).

On FFA (Figure 4), vascular occlusion can manifest as widespread arteriolar or branch retinal artery occlusion (BRAO) with severe retinal ischemia and NV [23]. Larger retinal vessels may be occluded leading to retinal and optic disc infarction that may also result in NV [31]. Central retinal artery occlusion (CRAO) and central retinal vein occlusion (CRVO), while very rarely seen in other causes of retinal vasculitis, have been reported secondary to SLE [32–34]. In one report involving 71 patients with SLE and retinal vasculopathy, three (6.3%) of the patients had either CRAO, CRVO, or ischemic optic neuropathy [35].

## 5. Antiphospholipid Syndrome

Antiphospholipid syndrome (APS) is an autoimmune disease characterized by the presence of vascular thrombosis, recurrent miscarriage, and antiphospholipid antibodies (IgG anticardiolipin, lupus anticoagulant, and anti-B_2_ glycoprotein-I antibody) [36]. Anticardiolipin antibody is associated with a higher incidence of occlusive vasculitis in the eye [37] and was reported to be present in 22.5% of patients with retinal vasoocclusive events in the absence of conventional risk factors of thrombosis [38].

**Figure 5:**
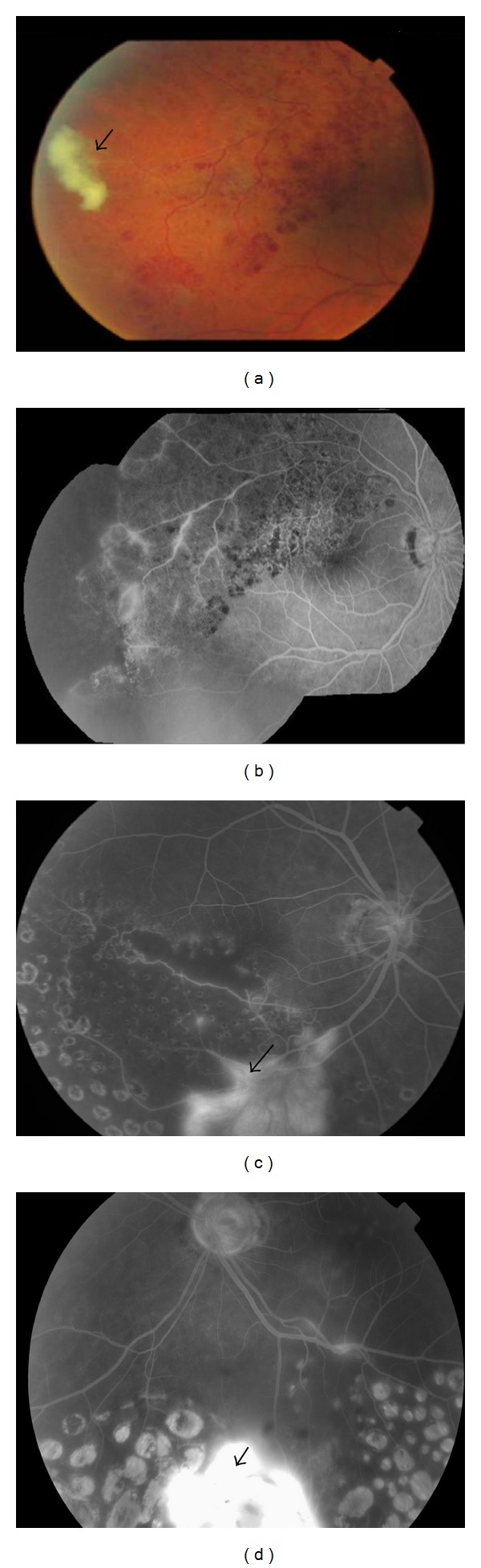
Fundus images show vasooclusion in patients with positive anticardiolipin antibodies. (a) Color image showing peripheral branch retinal vein occlusion with an intraretinal haemorrhage and peripheral fibrovascular tuft (Arrow). (b) Fluorescein angiography of the same patient showing vascular fluorescein leakage together with peripheral retinal nonperfusion. (c, d) Fluorescein angiography of another patient demonstrating bilateral retinal ischemia with areas of hypoperfusion covered with laser photocoagulation scars. There are also bilateral fibrovascular tufts leaking fluorescein (arrows).

APS can be associated with ocular manifestations, occurring in up to 80% of cases and can commonly result in retinal vasooclusion independent of the presence of SLE (Figure 5) [39]. APS can result in unilateral and bilateral CRVO, CRAO, BRVO, BRAO, and cilioretinal artery occlusion [40–42]. In rare occasions, nonarteritic anterior ischemic optic neuropathy has also been reported [43, 44]. It is not uncommon for patients to initially present with only ocular findings before the diagnosis of APS is established. Therefore, it is reasonable to exclude this condition in younger patients presenting with occlusive vasculitis in the absence of known systemic risk factors, allowing for early management and preventing further systemic manifestations associated with APS [45].

## 6. Sarcoidosis

**Figure 6:**
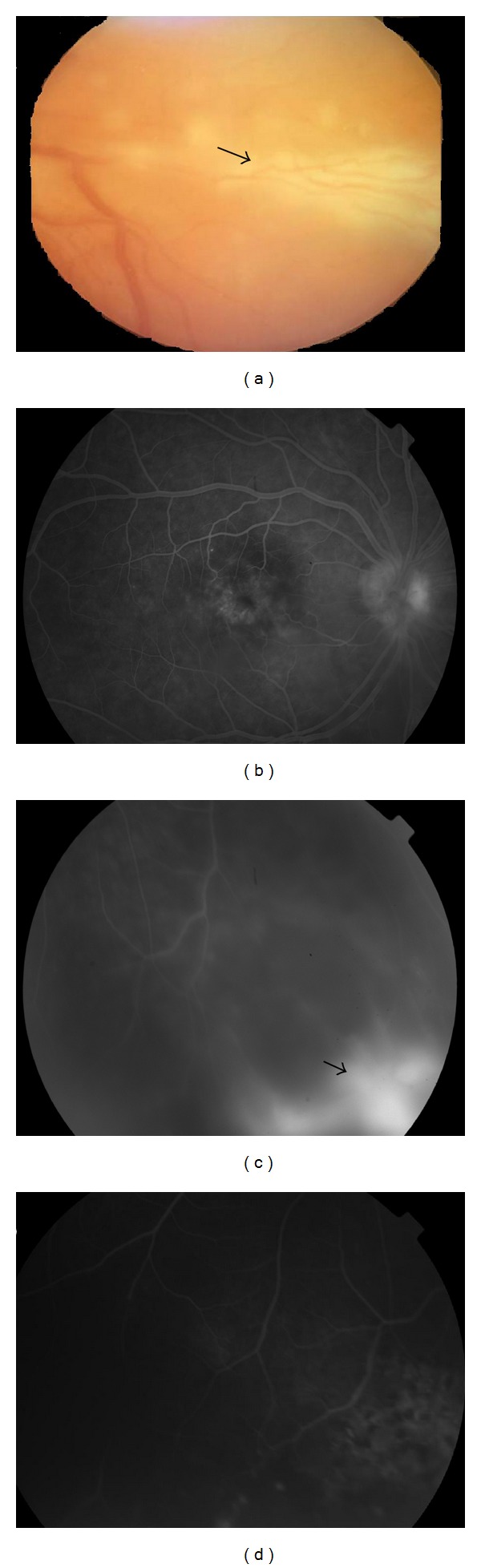
(a) Color fundus imaging of retinal vasculitis secondary to sarcoidosis showing perivenous exudates, “candle wax drippings.” (b) Fundus fluorescein angiography of an eye with ischemic vasculitis secondary to sarcoidosis shows leakage at macula secondary to macular edema; (c) peripheral retinal hypoperfusion with focal area of fluorescein leakage corresponding to new vessel formation (arrow). (d) Image taken five months following treatment with systemic corticosteroids and focal laser photocoagulation shows regression of the neovascularization.

Ocular involvement has been observed in 25–60% of patients with systemic sarcoidosis. In these cases, retinal vasculitis in the form of multifocal periphlebitis has been reported in 37% of patients with ocular sarcoidosis [35]. Retinal periphlebitis is a common ocular manifestation and was considered by the first International Workshop On Ocular Sarcoidosis as one of seven clinical signs that comprise the diagnosis of ocular sarcoidosis [46]. Although ocular sarcoidosis is typically associated with nonobstructive vasculitis, ischemic retinal vasculitis has rarely been reported in patients with sarcoidosis. Typical features of the involved vessels include segmental cuffing or extensive sheathing and perivenous exudates, known as “candle wax drippings” associated with vasculitis on FFA that mainly involves midperipheral retinal veins. Additional vascular features include the presence of macroaneurysms, peripheral vessel closure, and NV (Figure 6) [5, 47].

In a study including 75 eyes of patients with sarcoid related uveitis, 37% had retinal vasculitis, three of which had ischemic vasculitis associated with NV [48]. In another study involving 68 patients with posterior uveitis related to sarcoidosis, NVD and VH were reported in 4% of cases, with an increased incidence of VH up to 16% in the young age group [49]. Branch retinal vein occlusion (BRVO), although very rare, has been previously reported especially among young age group in the presence [50] or absence [51] of iridocyclitis. The exact underlying pathology of retinal vasculitis in these cases is not clear. One case report documented the presence of noncaseating granulomas around retinal blood vessels following a postmortem examination of a patient with known idiopathic ischemic retinal vasculitis. Even though such histological finding was suggestive of ocular sarcoidosis, there was no similar findings in the blood vessels elsewhere and no features of systemic sarcoidosis [52].

## 7. Multiple Sclerosis

The risk of uveitis in patients with multiple sclerosis (MS) is ten times higher compared to the general population, commonly in the form of intermediate uveitis [53]. However, the presence of peripheral periphlebitis was described in the early case reports of MS related uveitis [54, 55]. A review of 1254 uveitis case records at a tertiary eye centre in the United States found 14 (1.3%) to be MS related uveitis, with more than half of the cases associated with vasculitis [56]. Periphlebitis has been suggested to be a risk factor for the development of neurological manifestations of MS, including optic neuritis [57, 58].

Many theories have been proposed to explain the pathophysiological correlation between MS and the presence of periphlebitis [59]. In an autopsy series of 93 eyes from patients with an established diagnosis of MS, seven showed segmental perivenular infiltrates of lymphocytes and plasma cells [60]; lymphocyte and plasma cells were also concomitantly observed around retinal and central nervous system veins in two patients with MS, leading to the conclusion that periphlebitis is an early event that may lead to plaque formation in the brain [61].

**Figure 7:**
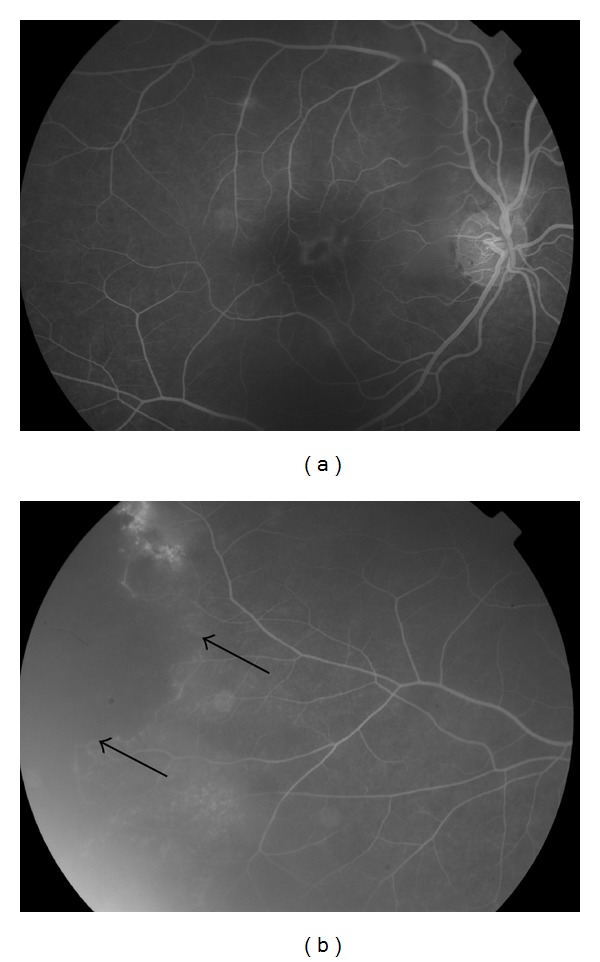
Fundus fluorescein angiography images of a right eye with intermediate uveitis associated with multiple sclerosis; (a) shows fluorescein leakage at the macula secondary to macular edema and (b) peripheral capillary dropout (arrows).

While periphlebitis has been reported in 20% of eyes [62], occlusive vasculitis and NV (Figure 7) are rare complications in MS related uveitis [63–67]. In a case series of 16 patients with MS related uveitis, eight suffered from ischemic retinal vasculitis with NV requiring SLP, while three eyes had unresolved VH secondary to NV requiring vitrectomy [63]. Peripheral retinal ischemia can be severe and had been reported to cause bilateral rubeosis iridis and neovascular glaucoma. While the rubeotic vessels regressed following treatment with oral corticosteroids and SLP, one eye required trabeculectomy to manage the glaucoma. No steroid sparing drugs were required in this case [68]. Although the presence of VH in uveitis can be highly suspicious of ocular Behçet's or sarcoidosis, the presence of MS may also need to be excluded in patients with intermediate uveitis that develop VH. In a series of 25 patients with MS related intermediate uveitis, six (24%) had periphlebitis associated with retinal ischemia and VH and four had NV on angiography. VH occurred at an average of five years following onset of uveitis, while it was the initial presenting manifestation in two patients [64]. The visual prognosis of MS related uveitis is generally good [56]; however, in those with occlusive vasculitis and NV it may varies. In one report, two of six patients with retinal ischemia and VH had a final vision of 20/80 five years after onset of VH [64].

## 8. Other Causes of Occlusive Retinal Vasculitis

**Figure 8:**
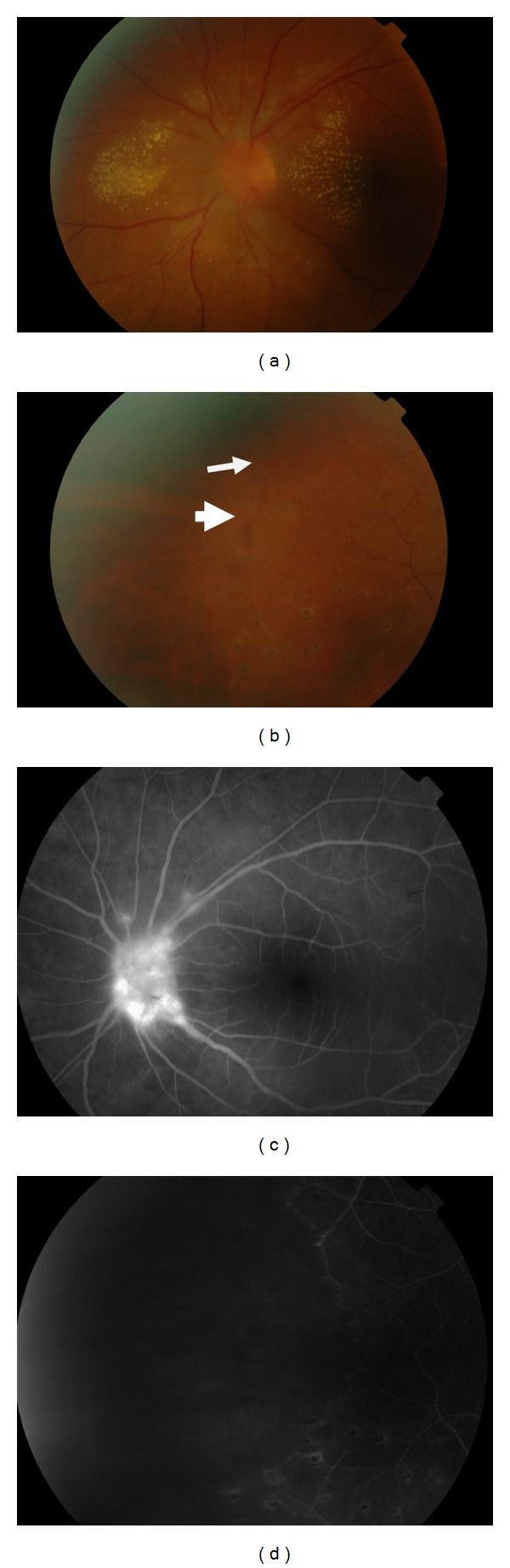
Fundus images of a patient with IRVAN syndrome. (a) Color image showing retinal exudates at the posterior pole involving the macula together with optic disc swelling and hyperemia. (b) Multiple pigmented chorioretinal lesions at the midperiphery (arrow head) together with evidence of vascular sheathing (arrow). (c) Fundus fluorescein angiography showing dye leakage at the optic disc. (d) Wide area of retinal nonperfusion.

Idiopathic retinal vasculitis, arteriolar macroaneurysms, and neuroretinitis (IRVAN) is characterized by recurrent multiple branch retinal arterial occlusions of unknown cause in one or both eyes of healthy middle-aged patients with no associated ocular or systemic etiology. An important cause of visual loss in IRVAN is chronic macular edema with hard exudate accumulation in the fovea (Figure 8). Vision loss also occurs secondary to peripheral capillary nonperfusion leading to NV and tractional retinal detachment [69].

Crohn's disease has been reported to be associated with ischemic retinal vasculitis, NV [70], neovascular glaucoma [71], and CRAO [72]. West Nile virus infection has been associated with chorioretinitis as its most common ocular finding, whereas occlusive retinal vasculitis is an uncommon finding reported to date in eight cases. Findings include perivascular sheathing, microaneurysms, cotton wool spots, intraretinal hemorrhages, and NV with or without macular ischaemia. Interestingly, six of these cases with established West Nile virus infection also suffered from diabetes mellitus [73, 74].

## 9. Treatment

Management of vasculitis and associated vascular occlusion can be challenging as most complications can result in severe visual loss mainly secondary to macular edema, macular ischemia, and retinal detachment.

### 9.1. Systemic Immunosuppressant

Severe retinal vasculitis requires adequate inflammation control using corticosteroids and, in noninfectious vasculitis, may need the addition of IMS agents. BD with severe posterior segment involvement, including retinal vasculitis, is initially treated with a combination of corticosteroids and IMS agents [75]. Cyclosporine A is effective and has long-term inflammatory control but can be associated with renal toxicity [76]. Meanwhile, azathioprine in BD with retinal vasculitis may not be very effective in producing complete resolution and relapse prevention during corticosteroid tapering [77]. In ocular sarcoidosis, the presence of retinal vasculitis requires the use of systemic corticosteroids and often the addition of IMS agents, most commonly methotrexate [78]. In SLE vasculopathy, systemic corticosteroids and IMS, such as cyclophosphamide and mycophenolate mofetil, are established treatments that can reduce vasculopathy and resolve cotton wool spots [79], though there is little evidence supporting their role in preventing the progression of retinal vasooclusion [23]. In presumed TB vasculitis, commencing systemic anti-TB therapy is useful in controlling the inflammation by suppressing the active TB focus, which causes immune activation and triggers uveitis. In addition, adjunctive use of systemic corticosteroid therapy may be required in the management of these cases to prevent damage to ocular tissues especially from delayed hypersensitivity.

### 9.2. Biologics

Antitumor necrosis factor alpha (TNF-*α*) drugs such as infliximab and adalimumab have been used successfully in the management of sight threatening retinal vasculitis. In severe ocular BD, anti-TNF-*α* can be considered as first-line IMS treatment [80] or used in cases refractory to other IMS to reduce the risk of severe visual loss and promote long term remission of uveitis [18, 81, 82]. Extended treatment with infliximab has also been effective in resolving NVD and improving visual outcome in retinal vasculitis secondary to BD [83, 84]. Anti-TNF-*α* is used successfully in treating refractive cases of sarcoidosis with retinal vasculitis, especially infliximab [85, 86] and adalimumab [87, 88]. Clinical reports on the use of infliximab to control ischemic retinal vasculitis secondary to sarcoidosis have shown good results, especially in cases where ocular symptoms manifest despite the use of IMS agents [89]. Meanwhile, etanercept is not only less effective in managing sarcoidosis but also reported to induce sarcoid intermediate and panuveitis [90, 91]. It should be noted that anti-TNF, often used in the management of severe noninfectious uveitis, should be avoided in treating MS related uveitis as it may precipitate or exacerbate nerve demyelination and worsen the neurological manifestations of this disease [92]. Infliximab used in patients with IRVAN was very successful in inducing dramatic resolution of ocular inflammation, reduction of retinal exudation, improving nerve leakage, and vision improvement after the first dose of infliximab therapy. However, it was not useful in preventing NV formation which occurred months later requiring laser therapy [93].

The use of rituximab, a chimeric monoclonal antibody against CD20+ B-cells, demonstrated some benefit in treating severe cases of SLE in uncontrolled studies but failed to prove superiority against placebo groups in a randomized controlled trial [94]. Rituximab combined with cyclophosphamide infusions was shown to result in rapid resolution of retinal vasooclusion in a pediatric group of SLE patients when used early in the course of the disease [95].

Interferon alfa (INF-*α*) therapies have been used in selected conditions to control inflammation. In ocular BD, INF-*α*-2a therapy was reported to provide long lasting remission in up to 55% of cases even after discontinuation of therapy [96]. In a retrospective study, INF-*α*-2a was effective in controlling retinal vasculitis in 36/38 eyes with BD and in 18/22 eyes with other causes of retinal vasculitis [97]. INF-*α*-2a may also result in reperfusion of vasooclusion [98] and induce NVD regression among BD vasculitis even in the absence of concomitant SLP [99]. In a retrospective review, five patients with BD and unilateral ischemic NVD received SLP and three had resolution of NVD following laser treatment while the other two patients responded only following additional treatment with INF-*α*-2a therapy [21].

The role of INF-*β*, an established treatment for MS, needs to be further studied to examine its effectiveness in controlling MS with retinal vasculitis. In a small retrospective study of 13 patients with MS related uveitis, ten of which were associated with retinal vasculitis, showed promising results with improvement of visual acuity in 71% of the eyes while a corticosteroid sparing effect was achieved in all cases [100].

### 9.3. Retinal Laser Photocoagulation and Intravitreal Anti-VEGF Injections

SLP is the main approach in managing NV that form secondary to occlusive vasculitis. In patients with presumed TB vasculitis, SLP was found to be very effective in inducing involution of NV. In a case series of 21 eyes with presumed TB vasculitis that received SLP for NV, there was no recurrence of VH or NV formation within a mean follow-up period of 18 months [12]. In BD, SLP is useful in inducing regression of NV and preventing further complications such as NV glaucoma [101]. In patients with IRVAN, SLP has been recommended in the presence of retinal ischemia before or shortly after the formation of NV regardless of the extent of vascular closure in order to prevent its progression and maintain good visual outcome [102]. Another study suggested using SLP only in eyes with retinal ischemia involving more than two quadrants [103]. In addition to SLP, other treatment options for IRVAN include macular grid laser, vitrectomy, and anti-TNF-*α* agents with a smaller role for corticosteroids [93, 104].

The primary treatment of retinal NV among patients with SLE and APS vasculopathy involves the use of SLP to the ischemic area with or without intravitreal anti-VEGF agents [40]. Unlike cases with presumed TB vasculitis, SLP is less effective in causing regression of NV in SLE and APS vasculopathy. In a systematic review of the literature, SLP performed on 22 eyes caused regression of the NV and stabilization of vision in only 54% of the cases [23]. Thus, it is not uncommon to see NV formation with subsequent VH and vitreoretinal traction even after retinal laser application [28]. In the absence of randomized clinical trials, it is difficult to assess the role of SLP alone in controlling NV due to the concomitant use of IMS medications in most cases. Intravitreal bevacizumab can be used in eyes with recurrent or persistent NV following SLP. A reported case of SLE with NVE that progressed despite the use of IMS and fill-in laser did respond to one intravitreal injection of bevacizumab resulting in NVE regression with no new bleeding over three months followup [105]. However, bevacizumab itself can reduce retinal perfusion and worsen retinal ischemia and therefore should be administered concomitantly with SLP. In a report of two patients with SLE, one received bevacizumab combined with SLP that resulted in halting the progression of the vascular occlusion with regression of the NVD. The second patient, who did not have laser, had progression of retinal ischemia with secondary NVE within a month of injecting bevacizumab [106]. In rare cases, intravitreal bevacizumab was reported to aggravate capillary nonperfusion within a day following injection despite previous administration of SLP [107].

### 9.4. Other Treatment Options

Plasma exchange has not shown any additional benefit in the management of nonocular manifestations of SLE and is only recommended for severe SLE crisis such as acute cerebritis or alveolar hemorrhage. However, in severe SLE cases, plasma exchange has been reported to show some benefit in stabilizing occlusive retinal vasculopathy when combined with rituximab infusion [108]. In another case report, plasma exchange combined with methotrexate was useful in providing rapid relief of symptoms but failed to provide a long term therapeutic benefit with a relapse of the vasculopathy six weeks after initiation of plasma exchange [109].

Catastrophic APS is treated using a combination of anticoagulants, corticosteroids, intravenous immunoglobulins, and plasma exchange, followed by prophylaxis with anticoagulant therapy [110]. Recurrence of thrombotic events in patients with APS is common. However, the role of prophylactic long-term anticoagulation therapy in preventing retinal vasoocclusive events is not well established, with a report of consecutive retinal vasooclusion occurring in a patient not on prophylaxis [41]. The role of such prophylaxis treatment in preventing recurrence of retinal vasoocclusive episodes should be addressed in prospective studies.

## 10. Conclusion

Patients with ischemic retinal vasculitis represent a significant management challenge and if not treated adequately it can lead to severe irreversible visual loss. The use of wide-field angiography should be encouraged in the diagnosis and monitoring of retinal vasculitis as it offers an advantage in detecting peripheral retinal ischemia and NV compared to traditional FFA imaging. Longitudinal or prospective studies are required to assess the effectiveness of IMS therapies in preventing the progression of occlusive retinal vasculitis and its complications.

## Figures and Tables

**Table 1:** Cause of retinal vasculitis according to the type of vessels involved and association with retinal ischemia.

	Mainly involve arteries	Mainly involve veins	Associated with retinal ischemia
Infectious disorders	Acute retinal necrosis Toxoplasmosis Cat scratch disease West Nile virus	Tuberculous hypersensitivity Syphilis CMV HIV Rift Valley fever virus HTLV-1	Tuberculous hypersensitivity West Nile virus

Noninfectious disorders	SLE APHA Takayasu's disease IRVAN GPA Churg-Strauss syndrome Crohn's disease Polyarteritis nodosa Susac syndrome Dermatomyositis	Behçet's disease Sarcoidosis Multiple sclerosis Birdshot chorioretinopathy APMPPE Pars planitis HLAB27 associated uveitis	Behçet's disease Sarcoidosis Multiple sclerosis SLE APHA Takayasu's disease IRVAN GPA Dermatomyositis Churg-Strauss syndrome Crohn's disease Polyarteritis nodosa Susac syndrome Idiopathic retinal vasculitis

SLE: systemic lopus erythematosus; APHA: antiphospholipid antibody syndrome; IRVAN: idiopathic retinal vasculitis, arteriolar macroaneurysms, and neuroretinitis; CMV: cytomegalovirus; HIV: human immunodeficiency virus; HTLV-1: human T-cell lymphoma virus type 1; APMPPE: acute posterior multifocal placoid pigment epitheliopathy; GPA: granulomatosis with polyangiitis.
